# Proceedings of the Regular Semi-Annual Meeting of the Brazos Valley Medical Association, May 10, 1898

**Published:** 1898-06

**Authors:** W. B. Briggs

**Affiliations:** Secretary


### Society Notes.

---

### Proceedings of the Regular Semi-Annual Meeting of the Brazos Valley Medical Association, May 10, 1898.

---

The Brazos Valley Medical Association convened at Casimer's opera house, Calvert, Texas, at 2 p. m., May 10, 1898.

Meeting called to order by President Dr. G. W Abney, invocation by Rev. James Kilgore.

The address of welcome on the part of the city of Calvert was delivered by the mayor, Hon. C. D. Meredith. This was followed by an address of welcome on the part of the citizens and the medical profession of Calvert, by Dr. Daniel Parker.

The response was made by Dr. I. P. Sessions, of Rockdale. The following papers were then read: "Indolent Ulcers," by Dr. Daniel Parker, of Calvert; "Metritis," by Dr. E. A. Harris, of Navasota; "Pneumonia," by Dr. I. P. Sessions, of Rockdale; "Dengue Fever, With Special Reference to its Diagnosis from Yellow Fever," by Dr. W. B. Briggs, of Easterly; "Entero Colitis," by Dr. W. S. Starkey, of Waco; "Hysteria," by Dr. R. S. Carroll, of Calvert; "Diphtheria," by Dr. Eugene Brittain, of Bremond; "Sore Eyes and Some Points on Differential Diagnosis," by Dr. E. P. Daviss, of Houston; "Report of Laparotomy, for Obstruction of the Bowels," by Dr. F. R. Collard, of Wheelock, Texas; "Gonorrhœa," by Dr. R. W.

Nobles, of Temple; *"The Need of and How to Secure Medical Legislation in Texas," by Dr. H. W. Cummings, of Hearne; "The Doctor as a Public Health Officer, and His Relation to the People He Represents," by Dr. G. R. Tabor, of Bryan; "Report of Cases," by Dr. J. C. Vannuys, of Franklin, Dr. J. M. Nicks, of Stone City and others.

The following officers were unanimously chosen for the ensuing year: President, Dr. Geo. R. Tabor, Bryan; First Vice-President, Dr. I. P. Sessions, of Rockdale; Second Vice President, Dr. J. C. Vannuys, of Franklin; Secretary, Dr. W. B. Briggs, of Easterly, re-election; Treasurer, Dr. J. W. Hudson, of Milano.

Judicial Committee—Dr. R. S. Glass, of Franklin; Dr. R. S. Carroll, of Calvert; Dr. J. M. Nicks, of Stone City; Dr. S. J. Emory, of Navasota, and Dr. Daniel Parker, of Calvert.

Orator—Dr. H. W. Cummings, of Hearne.

Thirteen physicians joined the Association.

Rockdale was selected for the place of the next meeting of the Association, and the time selected, third Tuesday and Wednesday in November next, the same being the 15th and 16th days of the month. Dr. I. P. Sessions was appointed chirman of the Entertainment Committee for the Rockdale meeting.

After adjournment came a trip to the coal mines, which was highly enjoyed.

The entertainment at the Columbia Club rooms, given by the ladies and citizens of Calvert, was grand, and the banquet was all the most fastidious appetite could desire.

The following resolutions by the appointed committee, Drs. H. W. Cummings and E. M. Abney, were by a rising vote unanimously adopted and ordered printed:

WHEREAS, The ladies, citizens and physicians of Calvert have by their courtesy, hospitality and association, added so much to the pleasure, comfort and entertainment of the members of this association during its stay among them, and

WHEREAS, We consider such hospitality and association one of the most important means of encouraging and promoting the interest of these meetings; therefore be it

*Resolved*, That we extend to the ladies and citizens of Calvert our most sincere thanks for the pleasure they rendered us, and

---

*Published in this issue of the JOURNAL.

that we extend assurances of our true appreciation to the profession of Calvert for their faithful work in promoting the welfare of the members' visit by their kind hospitality to us.

*Resolved*, That we extend our hearty thanks to the managers of the excursion to the coal mines, to the Houston & Texas Central officials and to Mr. Beattie for his kindness in carrying us through the mine, in which all Robertson County should have a pride for such richness of our soil.

That we especially appreciate the musical, social and festival entertainment given us by the ladies and citizens of the Columbia Club rooms. W. B. BRIGGS, Secretary.

